# Supplementary material for: Factors influencing the adoption of e-government by female university students
Source: PLoS One. 2025 Jan 24;20(1):e0317685. doi: 10.1371/journal.pone.0317685 (PMC11760642; doi:10.1371/journal.pone.0317685)
Supplement: Attachment — Submitted filename: Review Report-PONE.pdf [file pone.0317685.s001.pdf]

Review Report: Factors influencing the adoption of e-government by female university students

1. It would be good to include the recent development of e-government by including the citations from 2023 to 2024. Why it is important to conduct a study of e-government? What is the research gap, motivation of study and contribution of this study? Why actual use and intention to use are included concurrently in this study?
2. The determinants are perceived usefulness, perceived ease of use, attitude and subjective norm. In introduction, include why these variables are selected and what are the differences of this study compared to other study? Why perceived behavioral control is not included in this study?
3. For research model and hypotheses, include the recent review of the literature such as in year 2023 and 2024 for each of the variable/construct. Further discuss the underpinning theories of the research framework. For H7, write more about the perceived connectedness and its relation to perceived ease of use.
4. H7. Perceived connectedness of smart home services has a positive effect on the perceived ease of use of the services. Suppose this hypothesis is H6? The proposed research framework in Figure 2 does not show this hypothesis. Please confirm if this hypothesis is tested in this study.
5. There are two H7. Please confirm if H7. Subjective norm has a positive influence on female university students' intention to use e-government services and H8. Intention to use has a positive influence on female university students' actual use of e-government system are supposed to be H7 and H8? Align the findings in Table 4. Indicate H1 to H8 in Table 4.
6. There is a lack of description of the data collection process, for instance, the use of questionnaire? Online? Face to face?
7. What are the sampling techniques and how to determine the minimum sample size?
8. It is recommended that to include the source of instruments/ measurement items in a table or describe it for each of the constructs.
9. What are the procedures used to analyze the data? Further discuss why PLS-SEM is selected as the estimation procedure. Did common method bias check is performed?
10. For structural model results presentation, include the interpretation of each of the hypothesis. Indicate which hypothesis is supported and not supported. Align Table 4 with Figure 2 as well as the hypotheses proposed in the section of 'research model and hypotheses'.
11. For discussion, include the justification for each of the hypothesis. For instance, H1 Perceived usefulness has a positive influence on female university students' attitude towards the use of e-government services. Indicate how does perceived

usefulness is related to attitude among female, supported by which previous study. Same applies to H2 to H8. Kindly cross check and align the hypothesis across whole manuscript.

12. What are the theoretical and practical implications?
13. Include limitations and recommendation for future study after the conclusion.
