# Supplementary material for: Factors influencing the adoption of e-government by female university students
Source: PLoS One. 2025 Jan 24;20(1):e0317685. doi: 10.1371/journal.pone.0317685 (PMC11760642; doi:10.1371/journal.pone.0317685)
Supplement: Attachment — Submitted filename: Response to Reviewers.docx [file pone.0317685.s002.docx]

Medellín, October 24, 2024

PLOS ONE editorial team

Best regards

Thank you for the opportunity to review the article. We accepted the referees' comments and made an effort to resolve their doubts and concerns. The comments helped us to improve the quality of the article and enriched the discussion about it, offering points of view that we had not considered in the initial version. The following changes were made, correctly marked with red letters and change control in the article:

| **Reviewer** | **Comment** | **Response** |
| --- | --- | --- |
| Reviewer 1 | Research Problematization and Contributions: The research problem and contributions need to be presented in a clearer and more concise manner. Clearly articulating the gap in the literature that your study addresses and the unique contributions your research makes will enhance the paper's overall impact. | The research problem and the contribution of the study are stated concisely in one paragraph. Also, the literature gap and the unique contributions of the study are presented. |
| Reviewer 1 | Hypotheses Development: The proposed hypotheses should be strengthened and must be theoretically supported. Consider providing a more robust rationale for each hypothesis, drawing on relevant theoretical frameworks and empirical evidence. | Research hypotheses are reinforced with recent literature (2023-2024). |
| Reviewer 1 | Literature Review and Theoretical Foundation: The literature review can be significantly strengthened by incorporating recent and relevant literature. This includes, but is not limited to, the following sources:  "Navigating the Roadmap to Meta-Governance Adoption" (DOI: 10.1108/GKMC-02-2024-0105)  "The Government Metaverse: Charting the Coordinates of Citizen Acceptance" (DOI: 10.1016/j.tele.2024.102109)  Retesting the TAM Model: The paper attempts to retest the Technology Acceptance Model (TAM), which has been extensively tested in numerous studies. As per the recommendations of Venkatesh, the retesting of TAM and UTAUT in marginally different contexts does not add substantial value to the ongoing discussion on technology adoption. It is recommended that you either justify the retesting in this context more convincingly or consider exploring a different or modified model that offers novel insights. | The studies recommended by the evaluator are included. The justification of the hypotheses is strengthened with recent references (2023-2024). With respect to the choice of TAM as a theory, as authors, we consider it appropriate for the objective of the study given its focus on emerging economies and underrepresented populations, such as young women in Medellin, Colombia, which offers a valuable and understudied perspective. Nevertheless, we recognize that the observation is valuable, so it is included within the limitations of the study. |
| Reviewer 1 | Discussion Section: The paper must include a detailed discussion section that elaborates on the main findings. This should be followed by a thorough exploration of both the theoretical and practical implications of your research. Doing so will help to contextualize your results within the broader field and provide clear guidance for future research and practice. | The discussion section is expanded and the theoretical and practical implications of the study are highlighted. |
| Reviewer 1 | Conclusion and Research Limitations: The paper should conclude with a strong conclusion that summarizes the key findings. Additionally, it is important to address the limitations of your study and propose directions for future research. This will not only demonstrate the rigor of your work but also guide others in the field. | The recommendation is followed |
| Reviewer 2 | 1. It would be good to include the recent development of e-government by including the citations from 2023 to 2024. Why it is important to conduct a study of e-government? What is the research gap, motivation of study and contribution of this study? Why actual use and intention to use are included concurrently in this study? | Recent literature (2023-2024) is included and the evaluator's questions are answered in the introduction section. |
| Reviewer 2 | 2. The determinants are perceived usefulness, perceived ease of use, attitude and subjective norm. In introduction, include why these variables are selected and what are the differences of this study compared to other study? Why perceived behavioral control is not included in this study? | The selection of variables is justified in the introduction. Also, the difference of this study from those that have been conducted previously is presented. This study focuses on the original TAM variables, which does not include behavioral control, since its focus is on the perception of the technology and not on the external barriers that may limit behavior. |
| Reviewer 2 | 3. For research model and hypotheses, include the recent review of the literature such as in year 2023 and 2024 for each of the variable/construct. Further discuss the underpinning theories of the research framework. For H7, write more about the perceived connectedness and its relation to perceived ease of use. | Recent theoretical references (2023-2024) are included in the justification of the hypotheses. Regarding the relationship between perceived ease of use and perceived connectivity, no theoretical information was included since it is not a factor that was taken into account in the theoretical model and was a variable that was mistakenly left out of the final version. We apologize for the error. |
| Reviewer 2 | 4. H7. Perceived connectedness of smart home services has a positive effect on the perceived ease of use of the services. Suppose this hypothesis is H6? The proposed research framework in Figure 2 does not show this hypothesis. Please confirm if this hypothesis is tested in this study. | Regarding the relationship between perceived ease of use and perceived connectivity, no theoretical information was included since it is not a factor that was taken into account in the theoretical model and was a variable that was mistakenly left out of the final version. We apologize for the error. |
| Reviewer 2 | 5. There are two H7. Please confirm if H7. Subjective norm has a positive influence on female university students’ intention to use e-government services and H8. Intention to use has a positive influence on female university students’ actual use of e-government system are supposed to be H7 and H8? Align the findings in Table 4. Indicate H1 to H8 in Table 4. | The error regarding the numbering of the hypotheses is corrected. The numbering is also included in table 4. |
| Reviewer 2 | 6. There is a lack of description of the data collection process, for instance, the use of questionnaire? Online? Face to face? | More information on the data collection process is provided in the methodology section. |
| Reviewer 2 | 7. What are the sampling techniques and how to determine the minimum sample size? | The type of sampling used is explained. |
| Reviewer 2 | 8. It is recommended that to include the source of instruments/ measurement items in a table or describe it for each of the constructs. | A table is added with information on the factors used and their respective indicators. The source is also added. |
| Reviewer 2 | 9. What are the procedures used to analyze the data? Further discuss why PLS-SEM is selected as the estimation procedure. Did common method bias check is performed? | The selection of the PLS-SEM approach is justified and the procedure used to verify the bias of the common method is explained. |
| Reviewer 2 | 10. For structural model results presentation, include the interpretation of each of the hypothesis. Indicate which hypothesis is supported and not supported. Align Table 4 with Figure 2 as well as the hypotheses proposed in the section of ‘research model and hypotheses’. | The hypotheses are ordered as proposed in the conceptual model, including characterization of the hypotheses that are fulfilled and those that are not, and aligned with Table 2. |
| Reviewer 2 | 11. For discussion, include the justification for each of the hypothesis. For instance, H1 Perceived usefulness has a positive influence on female university students’ attitude towards the use of e-government services. Indicate how does perceived usefulness is related to attitude among female, supported by which previous study. Same applies to H2 to H8. Kindly cross check and align the hypothesis across whole manuscript. | The discussion is complemented with the contrast with other studies that support the findings of the hypotheses of the model. Each hypothesis (e.g., H1) is highlighted to facilitate the identification of the contrast being made. |
| Reviewer 2 | 12. What are the theoretical and practical implications? | The theoretical and practical implications of the study are highlighted with a subtitle. |
| Reviewer 2 | 13. Include limitations and recommendation for future study after the conclusion. | The recommendation is followed. |

Thank you in advance for considering the article for your prestigious journal,

Sincerely yours,

The Authors
